# Microstructure Simulation and Constitutive Modelling of Magnetorheological Fluids Based on the Hexagonal Close-packed Structure

**DOI:** 10.3390/ma13071674

**Published:** 2020-04-03

**Authors:** Jintao Zhang, Wanli Song, Zhen Peng, Jinwei Gao, Na Wang, Seung-Bok Choi, Gi-Woo Kim

**Affiliations:** 1 School of Mechanical Engineering and Automation, Northeastern University, Shenyang 110819, China; zhangjt@stumail.neu.edu.cn (J.Z.); 1800391@stu.neu.edu.cn (Z.P.); gaojinwei94@163.com (J.G.); wangn@me.neu.edu.cn (N.W.); 2 Department of Mechanical Engineering, Inha University, Incheon 22212, Korea

**Keywords:** microstructure, particle dynamics analysis, magnetorheological fluids, constitutive modeling, hexagonal close-packed structure

## Abstract

This paper presents a new constitutive model of high particles concentrated magnetorheological fluids (MRFs) that is based on the hexagonal close-packed structure, which can reflect the micro-structures of the particles under the magnetic field. Firstly, the particle dynamic simulations for the forces sustained by carbonyl iron powder (CIP) particles of MRFs are performed in order to investigate the particles chain-forming process at different time nodes. Subsequently, according to the force analyses, a hexagonal close-packed structure, which differs from the existing single-chain structure and body-cantered cubic structure, is adopted to formulate a constitutive model of MRFs with high concentration of the magnetic-responsive particles. Several experiments are performed while considering crucial factors that influence on the chain-forming mechanism and, hence, change the field-dependent shear yield stress in order to validate the proposed model. These factors include the magnetic induction intensity, volume fraction and radius of CIP particles, and surfactant coating thickness. It is shown that the proposed modeling approach can predict the field-dependent shear yield stress much better than the single-chain model. In addition, it is identified that the shear yield stress is increased as the particle volume fraction increases and surfactant coating thickness decreases. It is believed that the proposed constitutive model can be effectively used to estimate the field-dependent shear yield stress of MRFs with a high concentration of iron particles.

## 1. Introduction

Magnetorheological fluids (MRFs), a new type of intelligent material, is usually composed of base carrier fluid and magnetic particles that are dispersed in the base carrier fluid. The flow state of MRFs can be rapidly governed by the imposed magnetic field. Some advantages of MRFs, such as easy controllability, quick response, and reversible change, enable it to display its huge potential applications in diverse fields including automotive suspension system [1], self-powered magnetic-field sensor [2], and vibration control structures [3,4]. It is well known that the macroscopic properties of materials are determined by their microstructures. Thus, the changes in the microstructure of MRFs will directly affect its macroscopic performance. A large amount of research works has been reported regarding the performance and constitutive model of MRFs since it has been discovered in 1948 by Rabinow. The flow state of MRFs between two parallel fixed plates under the influence of a constant magnetic field perpendicular to the flow direction, and a constitutive model of incompressible MRFs were investigated in [5]. Jolly et al. have established a standard isolated-chain model that is feasible only when the direction of magnetic dipolar interaction of adjacent particles is along the applied magnetic field in view of the system consisting of infinite chains of particles in MRFs [6]. However, the volume fraction of magnetic particles in MRFs must be low enough to ensure the calculation accuracy, leading to the limitation of this model. Varela-Jiménez et al. proposed a semi-empirical constitutive model in view of the flow state transition of MRFs under the magnetic field in order to investigate MRFs with high volume fraction of magnetic particles [7]. It has been shown that this model properly reflects the relationship between the shear yield stress and magnetic induction strength, volume fraction of carbonyl iron powder (CIP) and other factors. Pan Sheng et al. deeply investigated the relationship between the shear yield stress and the strength of magnetic field, temperature, and composition of MRFs and then constructed a new shear stress model [8,9]. What is more, an optimum percentage of particles in the carrier fluid is obtained to improve efficiency [10]. As early as 1997, R. Tao et al. have mentioned that the arrangement of particles in MRFs might form a dense hexagon when the electric field and magnetic field are compatible [11].

On the other hand, several experimental works have been done to find the relationship between the shear yield stress and temperature at different shear rates under inhomogeneous magnetic field. Gao Chunfu addressed that most of the constitutive models of MRFs proposed so far have been formulated based on the theory of magnetic dipole moment, according to which the magnetic particles under the external magnetic field are regarded as magnetic dipoles. The classical theoretical models of magnetic dipole only usually take the influence of the adjacent magnetic chains on the target particle into account and can only analyze a single particle separately to obtain its magnetic dipole moment in the particle chain [12]. Taking the magnetic dipole theory as a foundation, Chen et al. calculated the interaction force between CIP particles, and then established the dipole boundary theoretical model that has been verified by experiments [13]. Ma et al. put forward a distance weighting factor model of the chain-structure utilizing the ampere molecular current hypothesis [14]. This model describes the interaction force between the magnetic particles, and the Monte Carlo method is adopted to simulate the microstructure of magnetic particles with the aim of exploring the influences of microstructure and macro-mechanics behaviors of materials. The simulating coordinates and magnetic moments of CIP particles are extracted in order to solve the shear stress value in theory. Enhanced magnetorheological performance of highly uniform magnetic carbon nano particles was explored by Seungae Lee et al. to investigate the influence of particle size on MR properties [15]. There is still a blank in the study of constitutive model for the chain structures of MRFs in high concentration considering the influence of magnetic particle chains on each other, as evident from the above literature survey from both theoretical and empirical aspects.

Consequently, the main technical contribution of this work is to propose a new constitutive model that is based on the hexagonal close-packed structure that can reflect the micro-structures of the particles in high concentrated MRFs. In order to achieve this goal, firstly the particle dynamics analyses of the forces sustained by CIP particles in MRFs are investigated, showing the motion model of CIP particles. Subsequently, the simulations of the chain-forming process of MRFs at different time nodes under imposed magnetic field are provided. According to the above force analyses, a hexagonal close-packed structure, which differs from the existing single-chain structure and body-centered cubic structure, is proposed, which takes the effects of adjacent chains under the aggregation of carbonyl iron particles into consideration [16]. Subsequently, the constitutive model of MRFs is established by the particle dynamics method that is based on the hexagonal close-packed structure. A series of experiments on the influencing factors, such as the magnetic induction intensity, volume fraction and radius of CIP particles, and surfactant coating thickness, are carried out in order to validate the proposed constitutive model. From the comparative work between the predicted and measured results, it is shown that the proposed modeling approach can be effectively applied to understand the chain structures of the magnetic particles and, hence, provides a guideline to develop high performance of MRFs.

## 2. Methods

It is necessary to analyze the forces on CIP particles and its influence factors, and some equations of motion need to be established, to accurately study the microstructure of MRFs. After performing the analyses of the forces and the motion state, the microstructure of MRFs under the magnetic field can be simulated and an appropriate microstructure model is provided.

### 2.1. Particle Dynamics Analysis

The complex microstructure of MRFs and influence factors on the evolution process are difficult to be observed by experiments. Numerical simulation technology can overcome these shortcomings. As a step to completely develop the numerical simulation, this work deals with particle dynamics investigation on the microstructure of MRFs after comprehensive considerations [17,18,19]. The particle dynamics method (PDM) is a new type of simulation that was developed from molecular dynamics combined with discrete element method, of which the essence is to analyze the forces and motion state of particles based on the Newton’s law. Meanwhile, the positions of all particles in the system are calculated and updated continuously with the changing time, and, at last, the stable microstructure of the particles system is obtained. The magnetized CIP particles are affected not only by magnetic force, but also by Brownian motion, repulsion force between CIP particles, gravity, buoyancy force of the base carrier fluid, and viscous resistance. The Brownian motion is overcome by CIP particles to form chains due to the large applied magnetic field, so Brownian motion can be neglected when the particle dynamics analysis is conducted. The gravity of CIP particles and the buoyancy force of base carrier fluid are also ignored because they are considerably smaller to the magnetic interaction force between magnetic dipoles under the action of magnetic field. Therefore, only magnetic force, viscous resistance, and repulsive force are considered in this work. There are two assumptions when the PDM is used; the magnetic particles are ideal sphere-shaped with the same size and the external imposed magnetic field is uniformly distributed.

#### 2.1.1. Magnetic Field Force

To a certain extent, the magnetic field formed by magnetic dipole *i* is affected by that formed by other magnetic dipoles, which is always ignored by some of the proposed approaches. The magnetic interaction force *F_i_*^m^ of CIP particles is given by [20]:

(1) $$F_{i}^{m}=\frac{3\mu_{0}m^{2}}{4\pi }\overset{N}{\underset{j\neq i}{\sum }}\frac{1}{r_{ij}{}^{4}}[(1-5\cos^{2}\sigma )\bar{r_{ij}}+2\cos\sigma \bar{z}]$$

where, *a* is the radius of CIP particles, *H* the magnetic field intensity, *r*_ij_ the center distance of the CIP particle *i* and *j*, which is not less than 2*a*, *σ* the deviation angle between the central line of particle *i* and *j* and the direction of the magnetic field, *μ_0_* is the vacuum susceptibility with a value of 4π × 10^−7^, *χ* the relative magnetic susceptibility of particles, $\bar{r_{ij}}$ the unit position vector of the particle *i* and *j*, and $\bar{z}$ three-dimensional the unit vector in the magnetic field direction, respectively.

#### 2.1.2. Repulsive Force

When the CIP particles move under the action of magnetic field, a collision generating repulsive force occurs between the particles. The repulsive force, which can refrain the particles from position overlapping, increases exponentially with increasing displacement of CIP particles during the collision. The concept of material parameter is introduced for quantifying the rapidity of this increase of repulsion force. The simulation focuses on the iteration of the particle position, which is not subject to the motion state of particle itself. Thus, the moment that is generated by the particle rotation is neglected in the analysis in order to simplify the calculation. A relatively concise expression for calculating the repulsion force $F_{i}^{r}$ is given by [20]:

(2) $$F_{i}^{r}=A\frac{3\mu_{0}m^{2}}{32\pi a^{4}}\underset{i\neq j}{\sum }\vec{\bar{r_{ij}}}\exp\left[-\zeta \left(\frac{r_{ij}}{2a}-1\right)\right]$$

where, *ξ* denotes the material parameter and *m* is the mass of the CIP particle. The direction of repulsion force is along the line of two colliding particles centers. Suppose that the CIP particles are rigid spheres without elastic-plastic deformation. The parameter *A* is given for expressing zero interaction force, when two colliding particles are just in contact.

At the same time, it is necessary to consider the collision between the vessel wall and the particles. The wall repulsion force $F_{i\left(\mathrm{wall}\right)}^{r}$ along the magnetic field direction is given by [21]:

(3) $$F_{i\left(\mathrm{wall}\right)}^{r}=\sum_{i\neq j}\frac{3QB^{2}}{8a^{4}}\{\exp[-\zeta (\frac{z_{i}}{2a}-1)]+\exp[-\zeta (\frac{L_{z}-z_{i}}{2a}-1)]\}$$

(4) $$Q=(4\pi a^{6}/\mu_{0}){\left[\chi /(\chi +3)\right]}^{2}B^{2}$$

where, (4) and *z*_i_ represents the ordinate of particle *i* in the simulant space, *L*_z_ is the coordinate length of *Z* axis in the simulant space. The equation of wall repulsion force perpendicular to the magnetic field is derived from Equation (3) by transforming the longitudinal coordinates of particles into horizontal coordinates and the longitudinal length into horizontal length, respectively.

#### 2.1.3. Viscous Resistance

The base carrier fluid of MRFs is deionized water and the viscous resistance $F_{i}^{d}$ of CIP particles from deionized water is generally described by the Stokes equation [22] when moving in the base carrier fluid without shear:

(5) $$F_{i}^{d}=-6\pi a\eta v$$

where *ν* is the motion velocity of CIP particles and *η* is the zero magnetic field viscosity of base carrier fluid. Under the magnetic field, MRFs undergoes the shear with a rate of *γ*. The shearing action is perpendicular to the direction *Z* of the applied magnetic field along the *X* direction. Subsequently, the viscous resistance $F_{i}^{v}$ of CIP particles from the base carrier is achieved from:

(6) $$F_{i}^{v}=-6\pi a\eta \left(v-z_{i}\gamma \bar{x}\right)$$

In the above equation, $\bar{x}$ represents the unit vector in the X direction of the simulant space.

### 2.2. Establishment of Motion Model

On the base of aforesaid analyses, it can be included that the movement of CIP particles in MRFs is comprehensively affected by many forces, among which the magnetic field force, the repulsion force and the viscous resistance of the base carrier fluid are the main important elements that affect the movement, the chain-forming process, and the microstructure of MRFs. The CIP particle is acted upon by the magnetic field force, the repulsion force, and the viscous resistance. Therefore, Newton’s second law is introduced to establish the kinetic equation of CIP particles without shear action [23], which is written as:

(7) $$m\frac{d^{2}a_{i}}{dt^{2}}=F_{i}^{m}+F_{i}^{r}+F_{i\left(\mathrm{wall}\right)}^{r}+F_{i}^{d}$$

where *t* is time and *a_i_* is the radius of CIP particle *i*. The kinetic equation of CIP particles under shear action is given by:

(8) $$m\frac{d^{2}a_{i}}{dt^{2}}=F_{i}^{m}+F_{i}^{r}+F_{i\left(\mathrm{wall}\right)}^{r}+F_{i}^{v}$$

Specially, in a rotating machinery, the CIP particles of MRFs are subjected to centrifugal force *F*_cf_, as follows:

(9) $$F_{\mathrm{cf}}=mv^{2}R$$

where, *R* is the distance between the rotating machinery and the particle center. Accordingly, the kinetic equation of particle *i* undergoing the shear action in the rotating machinery is expressed as:

(10) $$m\frac{d^{2}a_{i}}{dt^{2}}=F_{i}^{m}+F_{i}^{r}+F_{i\left(\mathrm{wall}\right)}^{r}+F_{i}^{v}+F_{\mathrm{cf}}$$

Thereby, the following equation of CIP particles without shear action can be derived from the kinetic Equation (7):

(11) $$m\frac{d^{2}a_{i}}{dt^{2}}+D\frac{da_{i}}{dt}=F_{i}^{m}+F_{i}^{r}+F_{i\left(\mathrm{wall}\right)}^{r}$$

where, *D* = 6 *πaη*. The time step used in the simulation is Δ*t*, the term on the right side of Equation (11) is a constant and the left side is integrated at an infinitesimal time step. Subsequently, the derivation of particles position change at the time step Δ*t* is given, as follows:

(12) $$\Delta s_{i}=\Delta t\frac{F_{i}^{m}+F_{i}^{r}+F_{i\left(\mathrm{wall}\right)}^{r}}{D}+m\frac{v_{0}}{D}\left({1-e}^{-\Delta t\frac{D}{m}}\right)$$

Δs*_i_* is the variation of particle position at time step Δ*t*, and *v*_0_ is the particle velocity at the beginning of time step Δ*t.* When Δ*t* is much smaller than *m/D*, it is deduced from Equation (12):

(13) $$\Delta s_{i}=\Delta t\frac{F_{i}^{m}+F_{i}^{r}+F_{i\left(\mathrm{wall}\right)}^{r}}{D}$$

## 3. Results and Discussion

### 3.1. Microstructure Simulation

The chain-forming simulation result of CIP particles is conducted via MATLAB to obtain the microstructure and configuration of MRFs. This simulation is performed over a range of conditions that are given in Table 1.

**Figure 1:**
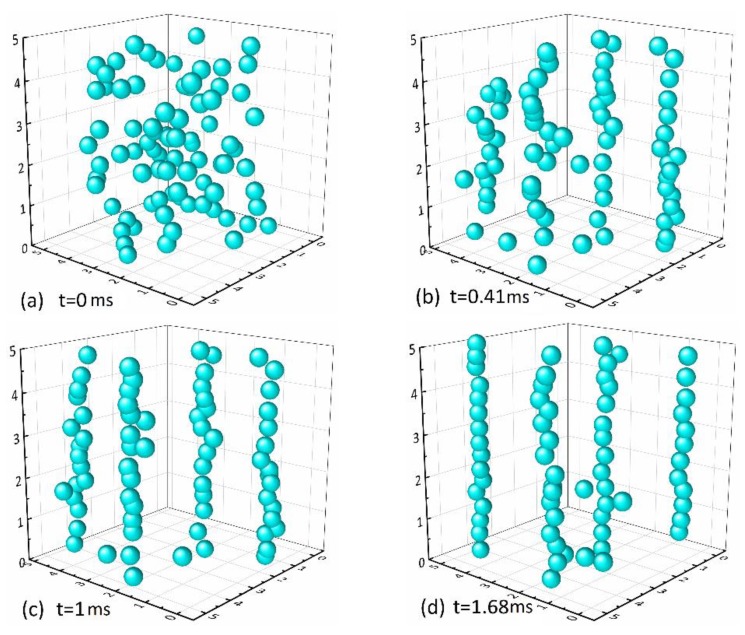
The three-dimensional distribution of the chain-forming process of magnetorheological fluid (MRF) at (**a**) 0 ms, (**b**) 0.41 ms, (**c**) 1 ms and (**d**) 1.68 ms under the magnetic field.

The CIP particles are randomly distributed in the base carrier fluid in the absence of magnetic field. The simulation starts with imposing the magnetic field on MRFs, and the CIP particles are rapidly magnetized into magnetic dipoles under the effects of magnetic force, repulsion force, and viscous resistance, collectively. The stress state of CIP particles is deduced based on the kinetic equations that were proposed earlier and then the displacement increment of CIP particles under constant iterations is figured out, according to which the steady state of the particle motion system is obtained, eventually. In this work, the volume fraction of CIP particles is set by 25%. The microstructure evolution of MRFs under magnetic field is simulated and the results are illustrated in Figure 1, the three-dimensional distribution of CIP particles position varying with time under magnetic field. Figure 1a shows the initial state of CIP particles with the random initial position given by the simulation system and zero initial velocity. Figure 1b–d is, respectively, the three-dimensional schematic diagrams of CIP particles distribution in MRFs under the external magnetic field at different time nodes, in which the unit of coordinate axis is meter.

The CIP particles are randomly distributed, as it appears from Figure 1a. At first, apply the magnetic field vertically downward along the Z-axis direction. Subsequently, the magnetized carbonyl iron particles attract each other and they begin to move under the synthetic influence of various forces of the magnetic force, viscous resistance, and buoyant force. The particles gradually arrange into short and medium chains. Randomly distributed particles get put into order by degrees. Since the microstructure of MRFs is essentially transformed, the corresponding macroscopic characteristics of MRFs are simultaneously changed from the liquid to the solid-like state. Short and medium branched chains and isolated chains in Figure 1b gradually aggregate with time into long chains, as shown in Figure 1c,d, which is consistent with the chain-forming process observed by previous experiments [24]. Besides, Figure 1d shows the arrangement and aggregation results of CIP particles at 1.68 ms after applying the magnetic field. It is observed that the particles finally form the chains configuration. As time goes on, the motion system of MRFs achieves the steady state. The microstructure dynamic analyses of MRFs and the results of chain-forming in the stable state are obtained, as previously discussed in this paper. In the following subsection, the microstructure model of MRFs under shear action in the steady state is discussed and the constitutive model of MRFs is established.

### 3.2. The Proposal for the Hexagonal Close-Packed Structure

The single-chain structure is mostly adopted in the incipient constitutive models of MRFs, with the merits of simple structure and strong versatility. However, in practical application, the volume fraction of CIP is generally high, and this results in the agglomeration of particle chains, which gets more serious with increasing volume fraction [18,25]. Therefore, it is inaccurate to describe the microstructure of a high concentration MRFs with the single-chain model, because it leads to the failure of MRFs constitutive model derived from this model to simulate the shear yield stress of MRFs appropriately. Some studies have been proposed in order to resolve the shortcomings of single-chain model. One example is the body-centered cubic structure model which can simulate high concentration MRFs. According to the body-centered cubic structure, the CIP particles are infinitely distributed in space in matrix arrangement. However, it is essential to build a more effective model due to the particularity that the universality of body-centered cubic structure is not high when the volume fraction of CIP varies.

**Figure 2:**
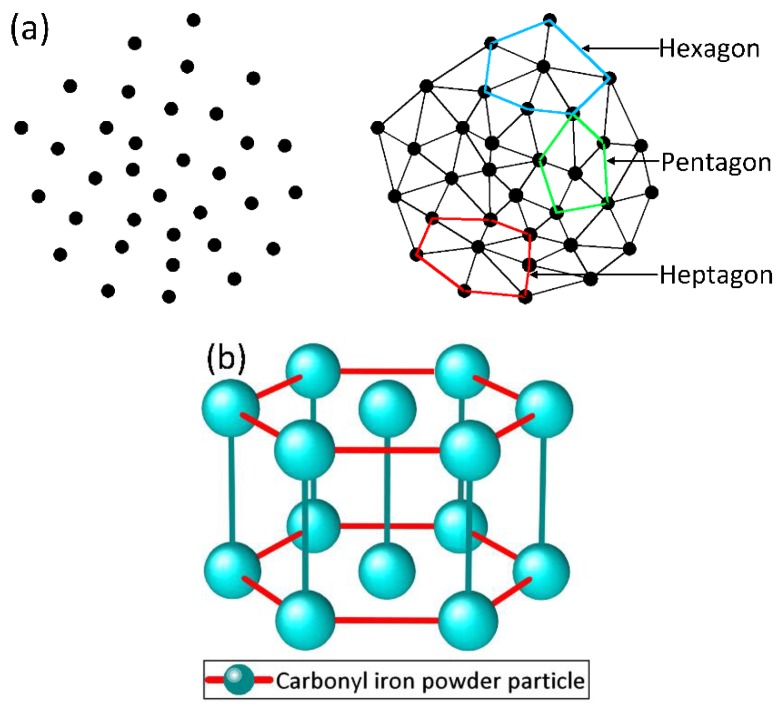
(**a**) The schematic of MRF microstructure perpendicular to magnetic field; (**b**) The schematic of the hexagonal close-packed structure.

It can be seen from Figure 2a that each point can be regarded as a chain of CIP particles. By triangulation, randomly distributed CIP particle chains in the direction perpendicular to the magnetic field forms pentagons, hexagons, or heptagons. Among which, the hexagons are the majority. This situation is more evident in high concentration MRFs. Accordingly, the irregular polygon is simplified to regular a hexagon to facilitate the calculation. Thus, in this work, the hexagonal close-packed structure is proposed. Figure 2b is the schematic drawing of the hexagonal close-packed structure. In this structure, each CIP chain has six adjacent chains and the distance between two chains is equal. The distance between adjacent chains is greatly affected by the volume fraction of CIP particles: the distance decreases with increasing volume fraction and it increases with decreasing volume fraction. In each chain, the magnetic dipole is impacted by other magnetic dipoles in this and adjacent chains. On this basis, the constitutive model of MRFs is to be constructed in this work. The salient feature of this method is to take a single chain as a unit [26] and then consider the influence of adjacent chains to adapt the hexagonal close-packed structure to the concentration change of MRFs.

### 3.3. Analyses of the Constitutive Model

In the previous studies, a single chain is separated from others to only discuss its own influence and ignore that from the adjacent chains. In this work, the effects of magnetic dipoles in the single chain and the adjacent chains on the single chain itself are considered through the hexagonal close-packed structure, and then a new constitutive model of MRFs considering the adjacent chains is formulated in order to resolve this problem.

#### 3.3.1. Force Analyses of Particles in a Single Chain

The dipole theory and the Maxwell stress tensor theory represent many studies on the constitutive model of MRFs [27,28], and it is known that the dipole theory is relatively concise and practical. Thus, this work builds the shear yield stress model of MRFs on account of it. The assumptions are made as followings: (1) the size and material of all CIP particles are the same; (2) the particles shape is approximately a sphere. With these premises, CIP particles are regarded as magnetic dipoles under the action of magnetic field. The magnetic energy model of a magnetic dipole in a single chain is constructed with the interaction between two magnetic dipoles into account. According to the magnetic dipole theory [29], the magnetic energy that is produced by arbitrary magnetic dipole *j* at *i* can be expressed, as follows:

(14) $$E_{ij}=\frac{\mu_{0}}{4\pi r_{ij}{}^{3}}\left[m_{i}m_{j}-3\left(\vec{r_{ij}}\cdot m_{i}\right)\cdot \left(\vec{r_{ij}}\cdot m_{j}\right)\right]$$

where *m_i_* is the magnetic dipole moment of the *i*-th magnetic dipole. The *m_i_* can be expressed, as follows:

(15) $$m_{i}=VM=\frac{4}{3}\pi a^{3}\chi H$$

where, *V* is the volume of a single CIP particle and *M* is the magnetization.

**Figure 3:**
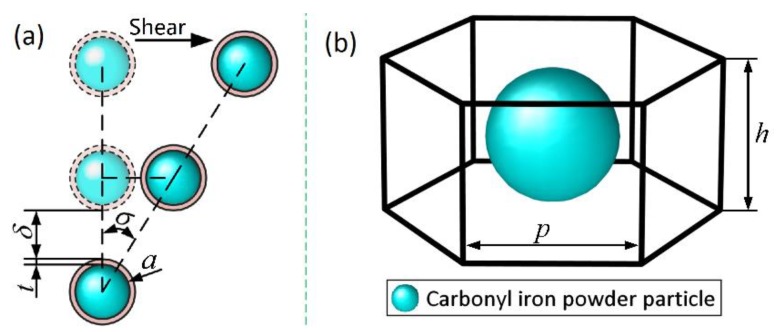
(**a**) The deviation angle of magnetic dipoles under shear in a single chain; (**b**) The space occupied by one single CIP particle.

Equation (14) indicates that the magnetic energy of the magnetic dipole *j* at a random position is inversely proportional to the third power of the distance from the magnetic dipole *i*. The interaction between magnetic dipoles is relatively strong along the magnetic field direction. Additionally, the force of the two interacting magnetic dipoles both in the same chain is greater than that in two different chains because the distance between the magnetic dipoles in the same chain is smaller than that in the adjacent chain. Suppose that there is an infinite amount of CIP particles in a single chain. Subsequently, by inserting Equation (15) into Equation (14) the magnetic energy for magnetic dipole *i* generated by other particles in this chain can be obtained by:

(16) $$E_{i1}=\overset{n}{\underset{k=1}{\sum }}\frac{\mu_{0}M^{2}V^{2}}{4\pi {\left[\frac{k\left(2a+2t+\delta \right)}{\cos\sigma }\right]}^{3}}\left(1-3{\left(\cos\sigma \right)}^{2}\right)=\frac{4C\pi a^{6}\mu_{0}\chi^{2}H^{2}}{9{\left(2a+2t+\delta \right)}^{3}}\left(1-3\cos^{2}\sigma \right)\cos^{3}\sigma$$

where, *n* is the number of magnetic dipoles in a single chain, *t* the surfactant coating thickness of a CIP particle, *δ* the net distance between the adjacent CIP particles in the same chain, and *σ* is the deviation angle of the magnetic dipole under shear, which are specifically shown in Figure 3a.

The influence coefficient of the magnetic dipole in the same chain is presented by the following equation:

(17) $$C=\overset{n}{\underset{k=1}{\sum }}\frac{1}{k^{3}}=1.2$$

#### 3.3.2. Analyses of the Influence from Adjacent Chains

For the constitutive model of high concentration MRFs, there is a need to consider the magnetic energy that is produced by the magnetic dipole not only at *i* in the same chain, but also in the adjacent chains. It is generally considered that the distance between the adjacent chains is greatly affected by the volume fraction of CIP particles. Taking the hexagonal close-packed structure as the basis, the distance *λ* between CIP particles chains can be deduced. The space occupied by a single CIP particle is a hexagonal prism, as shown in Figure 3b. Then, the volume ratio of the CIP particle to the hexagonal prism is the volume fraction *φ* of the CIP particles of MRFs, which is given by:

(18) $$\varphi =\frac{4\pi a^{3}}{3V_{L}}$$

where, *V*_L_ represents the volume of the hexagonal prism unit. At the same time, it can be seen that the height of the hexagonal prism is exactly *h*, and it follows that the volume of the hexagonal prism unit can be expressed, as follows:

(19) $$V_{L}=S_{L}h$$

**Figure 4:**
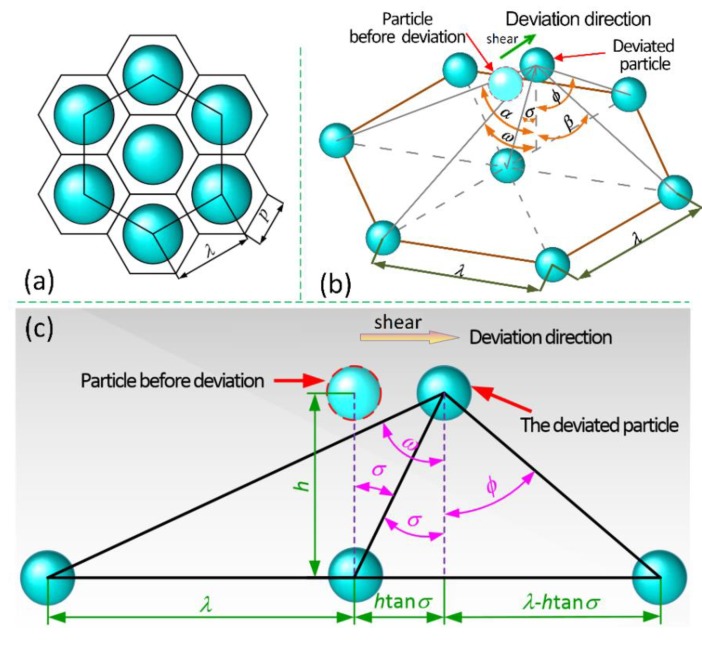
(**a**) The overall structure for the close-packed unit particles; (**b**) Angles between the offset magnetic dipole *i* and the contiguous magnetic dipoles in the vertical direction; and, (**c**) Angles between the front and rear magnetic dipoles and the offset magnetic dipole along shear direction.

Subsequently, the bottom area *S*_L_ of the hexagonal prism is obtained by solving the above two Equations (18) and (19). According to the top view of the hexagonal prism space occupied by CIP particles shown in Figure 4a, the following equation is achieved that contributes to deducing the side length *p* of the bottom of regular hexagonal prism:

(20) $$S_{L}=\frac{3\sqrt{3}}{2}p^{2}$$

Subsequently, the distance *λ* between CIP particle chains can be written as:

(21) $$\lambda =\sqrt{3}p=\sqrt{\frac{8\pi a^{3}}{3\sqrt{3}\varphi h}}$$

The central distance a between CIP particles in the same chain is taken as 2.2*a* in order to simplify the calculation. The ratio between dipolar and repulsion force of two particle chains along the magnetic field should not be too large or too small in order to obtain the optimum shear yield stress. *λ* should be bigger than 2.2*a* which is proved in previous studies and practical experience, because the ratio can reach the value 10 (0.1) when the distance between particle chains increases to *λ* / 2*a* = 1.1 (however, decreases to *λ*/2a = 0.9). [14,20]. The upper limit of volume fraction of the presented model comes out 45.4% by taking a as 2.2*a* into Equation (21). Moreover, the volume fraction of CIP particles in MRFs is usually less than this value due to the restriction of zero magnetic field viscosity and other conditions. Therefore, the proposed model meets the practical application conditions. It is verified from Equation (16) that the influence of magnetic dipoles in adjacent chains is much smaller than that in the same chain because the distance between magnetic dipoles in adjacent chains is larger than that in the same chain. Only the influence of the chains that directly adjoin to the chain of the magnetic dipole *i* is considered, while the influence of farther chains is ignored to predigest the constitutive model. The expression of magnetic energy of the magnetic dipoles in adjacent chains to the magnetic dipole *i* is derived by Equation (15), as follows:

(22) $$E_{i2}=\frac{4\pi a^{6}\mu_{0}\chi^{2}H^{2}}{9{\left(2a+2t+\delta \right)}^{3}}\left(1-3\cos^{2}\omega \right)\cos^{3}\omega$$

(23) $$E_{i3}=\frac{4\pi a^{6}\mu_{0}\chi^{2}H^{2}}{9{\left(2a+2t+\delta \right)}^{3}}\left(1-3\cos^{2}\phi \right)\cos^{3}\phi$$

(24) $$E_{i4}=\frac{8\pi a^{6}\mu_{0}\chi^{2}H^{2}}{9{\left(2a+2t+\delta \right)}^{3}}\left(1-3\cos^{2}\alpha \right)\cos^{3}\alpha$$

(25) $$E_{i5}=\frac{8\pi a^{6}\mu_{0}\chi^{2}H^{2}}{9{\left(2a+2t+\delta \right)}^{3}}\left(1-3\cos^{2}\beta \right)\cos^{3}\beta$$

where, *E*_i2_ and *E*_i3_ represent, respectively, the magnetic energy along the shear direction produced by the magnetic dipole, which is far from and closer to the magnetic dipole *i* after deviation under the shear action, *E*_i4_ and *E*_i5_ represent, respectively, the magnetic energy along the shear direction produced by the magnetic dipoles on both sides, which are far from and closer to the magnetic dipole *i* after deviation under the shear action. It is noted that the magnetic dipole only moves along the shear direction. As expressed in Figure 4b, *ω* and *ϕ* represent the angle between the offset magnetic dipole *i* and the far and close magnetic dipole in the vertical direction, while *β* and *α* separately denote the angle between the offset magnetic dipole *i* and the far and close magnetic dipoles on both sides in the vertical direction.

Figure 4c illustrates the angles between the front and rear magnetic dipoles and the offset magnetic dipole along the shear direction. To obtain those angles, the edge lengths of two right-angled sides of the right-angled triangle are first solved according to Figure 4c. Subsequently, applying the Pythagorean theorem yields the following result:

(26) $$\cos\omega =\frac{h}{\sqrt{{\left(\lambda +h\tan\sigma \right)}^{2}+h^{2}}}$$

Similarly, *ϕ* can be obtained:

(27) $$\cos\phi =\frac{h}{\sqrt{{\left(\lambda -h\tan\sigma \right)}^{2}+h^{2}}}$$

**Figure 5:**
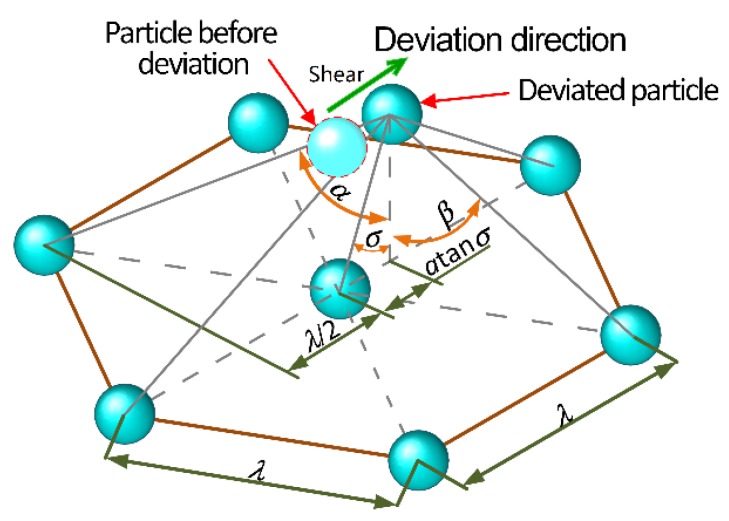
Angles between the magnetic dipoles on both sides and the offset magnetic dipole along shear direction.

Figure 5 describes the angles, respectively, between the magnetic dipoles on both sides and the offset magnetic dipole, and *β* and *α* are deduced based on the known conditions:

(28) $$\cos\beta =\frac{h}{\sqrt{{\left(\frac{\lambda }{2}-h\tan\sigma \right)}^{2}+\frac{3\lambda^{2}}{4}+h^{2}}}$$

(29) $$\cos\alpha =\frac{h}{\sqrt{{\left(\frac{\lambda }{2}+h\tan\sigma \right)}^{2}+\frac{3\lambda^{2}}{4}+h^{2}}}$$

#### 3.3.3. Constitutive Model of MRFs

As explained above, *E_i_* the total magnetic energy of magnetic dipole *i* is expressed as:

(30) $$E_{i}=E_{i1}+E_{i2}+E_{i3}+E_{i4}+E_{i5}$$

Subsequently, the magnetic energy that a single chain in unit length in the hexagonal close-packed structure has is solved as:

(31) $$E_{1}=\frac{n\cdot E_{i}}{L}$$

where, *L* represents the length of a single chain in a hexagonal closed-packed structure. The shear yield stress of the single chain is obtained by taking the derivative of Equation (31) [30]:

(32) $$\bar{\tau }=\frac{\partial E_{1}}{\partial \gamma }=\frac{\partial E_{1}}{\partial (\tan\sigma )}$$

Note that the shear yield stress of MRFs is comprised of the sum of shear yield stress that is formed by every single chain within the unit area [31]:

(33) $$\tau =N\bar{\tau }$$

(34) $$N=\frac{3\varphi V_{m}}{4\pi a^{3}n}\frac{L}{V_{m}}=\frac{3\varphi L}{4n\pi a^{3}}$$

where, *N* is the number of chains of CIP within unit area and *V*_m_ is the MRFs volume.

However, the CIP particles in this paper are hypothesized to be ideal, considering them to be equally sized and sphere-shaped and uniformly distributed in MRFs. However, the non-uniformity of CIP particles distribution and difference of the particle shape and size do exist, thus, the error that is caused by the non-uniformity of CIP particles distribution should be taken into consideration when the MRFs constitutive model is established. The parameter *k* is introduced to express the effect of in homogeneity of CIP particles on the shear yield stress of MRFs; the updated MRFs shear yield stress is as follows:

(35) $$\tau =\frac{4H^{2}a^{3}\varphi \chi^{2}\mu_{0}}{3k{\left(2a+2t+\delta \right)}^{3}}f\left(\sigma \right)$$

The complete form of Equation (35) is complicated and inconvenient, so, to simplify the expression, the simplified form is written as Equation (35). The function *f*(*σ*) in Equation (35) is derived in the Appendix A.

The unit of data measured of MRFs external magnetic field is magnetic induction intensity, which is substituted for the magnetic field intensity *H* in Equation (35), which is not measurable by instrument [32]. When considering the magnetization of paramagnetic particles, the magnetic induction intensity *B* is expressed by the following formula:

(36) $$B=\mu_{0}H\left(1+\chi \right)$$

The Froelicb–Kennelly material model [33] is adopted to write the relative magnetic susceptibility because of the nonlinear and saturated magnetization of CIP particles under magnetic field:

(37) $$\chi =\frac{\chi_{0}M_{d}}{\chi_{0}H+M_{d}}$$

In the above, *M*_d_ is the magnetization saturation of MRFs which has the relationship with the expression of *μ*_0_*Μ*_d_ = 2.1*φ*, *χ*_0_, in which the initial susceptibility value is 1000.

## 4. Experiments

The shear yield stress of MRFs is affected by many factors, as mentioned above. It can be seen that magnetic induction strength, size, and volume fraction of CIP particles and the surfactant coating thickness are the dominant influences on the shear yield stress of MRFs, according to the constitutive model of MRFs proposed. The following sections investigate the unknown parameters by the fitting method. In addition, the variable-controlling approach is used to explore these factors. Now some rules of experiments are set as follows:

- The magnetic induction intensity is 0.4 T.
- The volume fraction of CIP particles is 34%.
- Read the shear yield stress when the shear strain is 0.31.
- The relationship between the surfactant coating thickness and distance of particles is expressed as 2*t* + *δ* = 0.8 μm.
- The radius of all CIP particles is 4 μm.
- The temperature to conduct the experiments is 25 °C.

It is noted that, when the effect of a certain factor to the properties of MRFs is investigated, the other influenced factors are assumed to remain as unchanged according to the above settings.

### 4.1. The Preparation of MRFs for Experiment

MRFs is usually composed of additives and micron-sized magnetic particles that are uniformly distributed in water-based or oil-based carriers. The base carrier liquid is generally required to have a higher boiling point and lower freezing point to meet different application temperatures. The zero field viscosity of MRFs is usually greatly affected by the viscosity of base carrier which is generally proportional to its density, so the base carrier needs to have lower viscosity. When considering the sedimentation rate of the MRFs, the density of the base carrier should not be too low to avoid the density difference between the magnetic particles and the base carrier to be too large. In addition, in practical application, whether the oil-based or water-based carrier is chosen is on the basis of whether the material that the MRFs works on is water-soluble or oil-soluble. In practical application, many materials are water-insoluble and, according to Table 2, as below, water-based carrier fluid was selected for preparing the MRFs.

In the magnetic field, the rheological properties of MRFs mainly depend on the magnetic particles. The shear yield stress of MRFs is the macroscopic reflection of the force between the magnetic particles under the magnetic field. Therefore, the magnetic particles should possess high permeability and magnetic saturation strength to ensure the high shear yield stress of MRFs. At the same time, it is necessary to have a low hysteresis so that the MRFs can quickly change into Newtonian fluids after the external magnetic field disappears, and the viscosity can quickly return to zero-field-state viscosity for circulating the MRFs. Under comprehensive consideration, CIP, meeting the above requirements and its simple manufacture, is selected for preparing the MRFs as the magnetic particles. 

The solid magnetic particles usually disperse in the base carrier, but they are difficult to dissolve in the base carrier due to the large density difference between them, which is prone to cause the settlement of magnetic particles. Therefore, surfactant needs adding into MRFs to better the settlement stability. The surfactant in MRFs should have special molecular structure: one end is the functional group with high affinity for absorbing solid magnetic particles; the other end is the elastic group that is easily dispersed in the base carrier, which prevents the magnetic particles from approaching each other and agglomerating by space repulsion it produces, and at the same time reduces the sedimentation. The magnetic CIP particles in the MRFs are easy to be corroded and oxidized by the base carrier, which weakens the magnetic saturation strength of the magnetic particles. The surfactant coated on the surface of magnetic particles can effectively prevent it. Besides, the deionized water is chosen as base carrier mentioned above, thus sodium dodecyl sulfonate is selected as surfactant.

Table 3 summarizes the compositions of MRFs. So far, MRFs can be prepared according to the selected compositions above and Table 4.

### 4.2. Shear Stress-Strain Relationship

**Figure 6:**
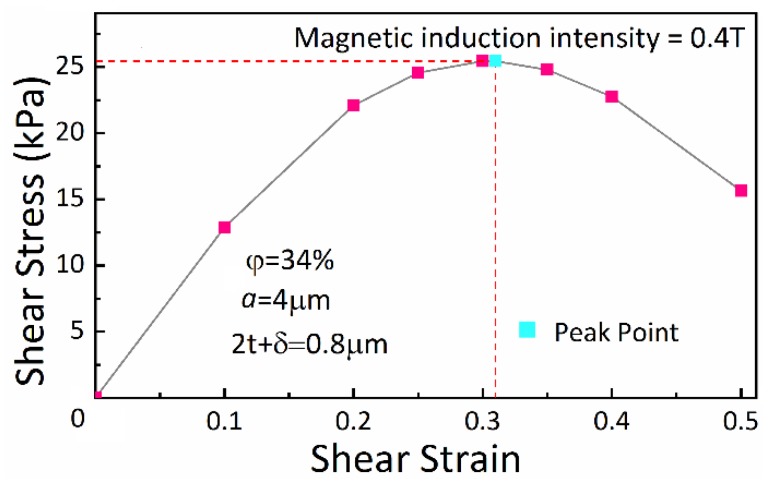
Shear stress-strain curve.

The shear yield stress of material is the maximum shear stress to resist deformation, which is reflected as the maximum stress point on the shear stress-strain curve. For MRFs, it is considered that the chain structure reaches its maximum deformation under the maximum shear stress. This group of experiment simulates the relationship between the shear stress and strain taking the strain as variable while the other factors unchanged. Figure 6 depicts the shear stress-strain relationship of MRFs in view of its chain structure. This result tells the point of maximum shear stress and the corresponding strain with a value of 0.31. The trend of the shear stress-strain relationship of other volume fraction of CIP particles, such as 25% and 40%, is also the same as the experimental result of 34%; the strain is almost often 0.31 when the shear stress reaches its maximum. Therefore, the shear stress is taken as the shear yield stress of MRFs in the subsequent experiments when the shear strain is 0.31.

### 4.3. Effect of Magnetic Induction Intensity

**Figure 7:**
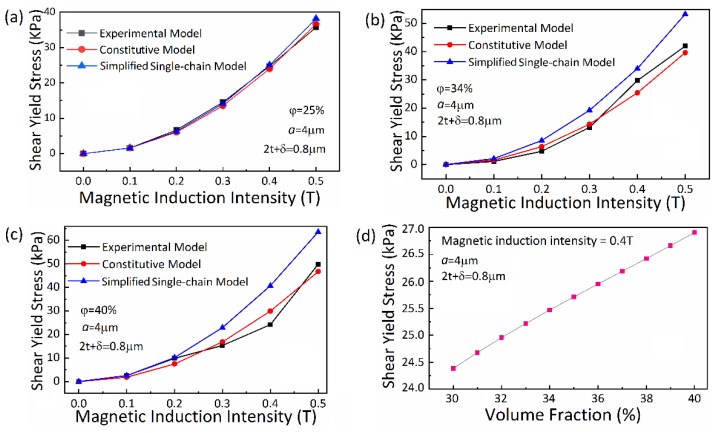
(**a**) Fitting diagram of shear yield stress and magnetic induction intensity relationship of known model. (*φ* = 25%); (**b**) and (**c**) Result of shear yield stress and magnetic induction intensity relationship of unknown model; and, (**d**) Result of shear yield stress and CIP volume fraction.

This group of experiment is designed not only to research the relationship between the shear yield stress and magnetic induction intensity, but also verify the strong adaptability of the constitutive model to the change of volume fraction of CIP particles. Thus, the groups of MRFs with different concentration are prepared to carry out this experiment and obtain the experimental data of MRFs shear stress at different concentration varying with the magnetic induction intensity. This group experiment is divided into three parts differing in the volume fraction of CIP particles. The magnetic induction intensity is taken as a variable in each group, while the other factors remain unchanged. Compare the experimental result with the Simplified Single-chain Model [34]. Figure 7a–c illustrate the curves of relationship between different volume fraction of CIP particles and magnetic induction intensity, respectively. In Figure 7, the ‘Experimental Model’ is obtained by fitting the experimental data that are measured in this work by conducting groups of experiments with MRFs prepared according to Section 4.1. The abscissa values are substituted into the constitutive model deduced above to obtain the corresponding ordinates, and the ‘Constitutive Model’ is formed by these points. The same is true for the ‘Simplified Single-chain Model’. Note that the ‘Simplified Single-chain Model’ is a simplified form, which definitely differs from the classic “Single-chain Model”, of force analysis of one single chain, according to the Section 3.3.1. It is simplified as compared with the “Constitutive Model”, which considers the effects of adjacent chains. Figure 7a is the fitting diagram of known model and Figure 7b is the simulation result of unknown model.

From Figure 7a–c, it is validated that the constitutive model constructed in this work well fits the trend of the shear yield stress as a function of the magnetic induction intensity. The saturation point of the shear yield stress moves backward with the increasing volume fraction of CIP particles, which is consistent with the previous research. Additionally, it is obviously seen from Figure 7b,c that the simulation of single-chain model to high concentration MRFs is not good showing relative larger error than the proposed constitutive model. It is remarked that the constitutive model that was built in this work can predict the shear yield stress well under magnetic saturation of MRFs, which is, in general, hard to achieve from experimental tests.

### 4.4. Effect of CIP Particles Volume Fraction

The volume fraction of CIP particles is taken as the variable while other parameters are kept to be unchanged to perform this group of experiment. Based on the constitutive model, the relationship between the shear yield stress and the volume fraction of CIP particles of MRFs is simulated and shown in Figure 7d. It is clearly observed that there is a linear relationship between shear yield stress and CIP volume fraction: the MRFs shear yield stress increases with the increasing CIP volume fraction, but the trend slowly becomes smooth, which means that the slope reduces. When the CIP particles deviate under the action of shear, the offset particles will approach to the CIP chain along the direction of deviation. According to the previous analyses of this work, at this moment, the force that is exerted on the offset particles by the chain along the deviation direction is greater than that exerted by the chain in reverse direction. Moreover, with the increasing CIP volume fraction, the distance between the particle chains decreases, which leads to the stronger influence of adjacent chains. Thus, the increasing trend of shear yield stress of MRFs decreases with increasing the volume fraction of the CIP particles.

### 4.5. Effect of CIP Particle Radius

**Figure 8:**
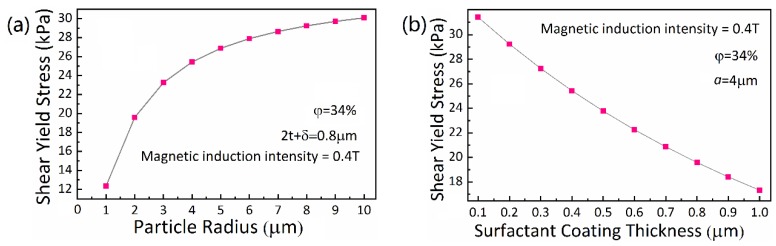
(**a**) Result of MRF shear yield stress of and CIP radius; (**b**) Result of shear yield stress and the surfactant coating thickness.

The effect of particle radius on the shear yield stress is studied in this group of experiments. With the fitting parameters determined and other conditions remaining unchanged, the changes of MRFs shear yield stress with CIP particle radius is simulated based on the model proposed and shown in Figure 8a. It is seen that the shear yield stress obviously increases with the increasing radius of CIP particles. Specifically, the magnetic interaction enhances with the increasing CIP particle radius, which leads to the increase of MRFs shear yield stress. At the same time, this trend is subjected to the increased center distance of particles, causing the diminished magnetic interaction. When the particle radius is relatively small, this increasing trend is more obvious; when the particle radius relatively is large, this trend slows down. The MRFs sedimentation stability is also affected by the changing radius of CIP particles, which is illustrated by the increase of sedimentation ratio with an increasing particle radius. Therefore, practically, the particle size should be carefully selected to meet the imposed requirements of MRFs, because a bigger particle size does not mean a better result of the properties of MRFs.

### 4.6. Effect of Surfactant Coating Thickness

Generally, the layer of surfactant coated on the CIPs is taken as even and the thickness of surfactant coating is regarded as an invariant for simplifying the calculation and facilitating the simulation [35]. In fact, with the increase of the surfactant volume fraction, the thickness of surfactant-coated particles increases. It is noted that when the surfactant volume fraction increases to a certain level, the coating thickness will stop increasing and the rest surfactant will have a negative influence on the properties on MR fluids. Therefore, an optimum volume fraction of surfactant is of great significance. The coating thickness is used to express the amount of surfactant that is adsorbed on or around the CIP particles. In this group of experiments, in the circumstances of other variables keeping invariant, the surfactant volume fraction is enhanced in order to thicken the surfactant coating. Subsequently, the thickness change of surfactant coating with MRFs shear yield stress is simulated and shown in Figure 8b. It is seen that the shear yield stress slowly decreases with an increasing surfactant coating thickness. This is attributable to the increscent distance between CIP particle centers that results from the increase of surfactant coating thickness. Although the surfactant takes up a small proportion in MRFs, it has great influence on the sedimentation stability. The sedimentation ratio of particles in MRFs decreases with the increase of surfactant coating thickness, which further leads to the reduction of MRFs shear yield stress. Through practical experience, the surfactant coating of CIP particles after reaching a certain thickness usually no longer increases. Therefore, the surfactant volume fraction should be selected based on that of the CIP particles, not being too large.

## 5. Conclusions

In this research, a constitutive model for predicting the shear yield stress of high concentrated MRFs has been proposed and its effectiveness is validated through comparative works with a single-chain model and experimental result. The microstructure of the particle chains of MRFs with high concentration was modeled by choosing the hexagonal close-packed structure. In the validation of the proposed model, magnetic induction intensity, volume fraction, size of CIP particles, and surfactant coating thickness affecting shear yield stress were adopted in order to investigate the influencing weight to the properties of MRFs. When one experimental group is under testing, the other factors have been kept to be unchanged to accurately observe the effect trend of each factor. It has been demonstrated through both simulation and experiment that the proposed constitutive model can predict the shear yield stress of high concentrated particles based MRFs much better than the single-chain model, which has been conventionally and frequently used so far. In other words, the presented constitutive model highly coincides to the experimental results showing a very similar trend of the shear yield stress of MRFs. 

It should be asserted that the proposed model based on the hexagonal close-packed structure of CIP chains is simple and novel. In addition, the constitutive model that is presented in this work provides a qualitative tool for readers to understand the microstructure and mechanical behavior of CIP particle chains in MRFs with high concentration. However, the model itself still exists complexity in part with several ideal assumptions that are made in the process of mathematical modeling. Therefore, some modifications of the proposed model with practical conditions are required to enhance the prediction accuracy and provide a theoretical basis for the application products of MRFs.

## Figures and Tables

**Table 1:** Time step and main parameters of carbonyl iron powder (CIP) particles in the simulation.

Properties	Parameters
Time step *Δt*	4.2 × 10^−8^ s
CIP particles density *ρ*	7860 kg·m^−3^
Viscosity of base carrier *η*	0.001 Pa·s
Vacuum permeability *μ*_0_	4π × 10^−7^ N·A^−2^
Material parameters *ζ*	30
Magnetic susceptibility of particles *χ*	1
Particle radius *a*	4 μm
External magnetic field *H*	200 kA/m

**Table 2:** Main physical properties of the base carrier.

Properties	Silicone Oil	Kerosene	Deionized Water
Density (g/cm^3^)	1.025–1.055	0.8	1
Boiling point (°C)	130–400	110–280	100
Freezing point (°C)	−50	−10	0
Viscosity (μPa·S)	0.6–3	0.5–0.6	1.01–1.02
Volatility	No	Volatilize	No
Toxicity	No	mild-toxicity	No

**Table 3:** Compositions of MRFs.

Composition	Material
Magnetic particle	CIP
Base carrier	Deionized water
Surfactant	Sodium dodecyl sulfonate

**Table 4:** Composition of MRFs in different groups.

Parameter	Group 1	Group 2	Group 3
Radius of CIP particles (μm)	4	4	4
Volume fraction of CIP particles (%)	25	34	40
Volume fraction of deionized water (%)	72	63	57
Volume fraction of surfactant (%)	3	3	3

## Abbreviations

MRFs	Magnetorheological fluids
PDM	Particle dynamics method
CIP	Carbonyl iron powder

## Appendix A

$\begin{array}{l} f(\sigma )\\ =-\frac{108.27\left(0.74\sqrt{\frac{1}{\varphi }}-2.2\tan\sigma \right)}{{\left(4.84+\frac{1.65}{\varphi }+{\left(0.74\sqrt{\frac{1}{\varphi }}-2.2\tan\sigma \right)}^{2}\right)}^{7/2}}\\ +\frac{11.18\left(1-\frac{14.52}{4.84+\frac{1.65}{\varphi }+{\left(0.74\sqrt{\frac{1}{\varphi }}-2.2\tan\sigma \right)}^{2}}\right)\left(0.74\sqrt{\frac{1}{\varphi }}-2.2\tan\sigma \right)}{{\left(4.84+\frac{1.65}{\varphi }+{\left(0.74\sqrt{\frac{1}{\varphi }}-2.2\tan\sigma \right)}^{2}\right)}^{5/2}}\\ -\frac{54.13\left(1.48\sqrt{\frac{1}{\varphi }}-2.2\tan\sigma \right)}{{\left(4.84+{\left(1.48\sqrt{\frac{1}{\varphi }}-2.2\tan\sigma \right)}^{2}\right)}^{7/2}}+\frac{5.59\left(1-\frac{14.52}{4.84+{\left(1.48\sqrt{\frac{1}{\varphi }}-2.2\tan\sigma \right)}^{2}}\right)\left(1.48\sqrt{\frac{1}{\varphi }}-2.2\tan\sigma \right)}{{\left(4.84+{\left(1.48\sqrt{\frac{1}{\varphi }}-2.2\tan\sigma \right)}^{2}\right)}^{5/2}}\\ +\frac{0.57\tan\sigma -0.29\tan\sigma \left(-2+\tan^{2}\sigma \right)}{{\left(\sec^{2}\sigma \right)}^{7/2}}+\frac{108.27\left(0.74\sqrt{\frac{1}{\varphi }}+2.2\tan\sigma \right)}{{\left(4.84+\frac{1.65}{\varphi }+{\left(0.74\sqrt{\frac{1}{\varphi }}+2.2\tan\sigma \right)}^{2}\right)}^{7/2}}\\ +\frac{54.13\left(1.48\sqrt{\frac{1}{\varphi }}+2.2\tan\sigma \right)}{{\left(4.84+{\left(1.48\sqrt{\frac{1}{\varphi }}+2.2\tan\sigma \right)}^{2}\right)}^{7/2}}-\frac{11.18\left(0.74\sqrt{\frac{1}{\varphi }}+2.2\tan\sigma \right)\left(1-\frac{14.52}{4.84+\frac{1.65}{\varphi }+{\left(0.74\sqrt{\frac{1}{\varphi }}+2.2\tan\sigma \right)}^{2}}\right)}{{\left(4.84+\frac{1.65}{\varphi }+{\left(0.74\sqrt{\frac{1}{\varphi }}+2.2\tan\sigma \right)}^{2}\right)}^{5/2}}\\ -\frac{5.59\left(1.48\sqrt{\frac{1}{\varphi }}+2.2\tan\sigma \right)\left(1-\frac{14.52}{4.84+{\left(1.48\sqrt{\frac{1}{\varphi }}+2.2\tan\sigma \right)}^{2}}\right)}{{\left(4.84+{\left(1.48\sqrt{\frac{1}{\varphi }}+2.2\tan\sigma \right)}^{2}\right)}^{5/2}} \end{array}$

## References

[1] Wang S.Y., Song W.L., Li H.L., Wang N. (2018). Modeling and multi-field simulation annlysis of a multi-cylindrical magneto-rheological brake. Int. J. Appl. Electrom..

[2] Qi S., Guo H.Y., Chen J., Fu J., Hu C.G., Yu M., Wang Z.L. (2018). Magnetorheological elastomers enabled high-sensitive self-powered tribo-sensor for magnetic field detection. Nanoscale.

[3] Zhang J.Q., Zhang J., Kong Y.N., Gao Y.Q., Jia J.F., Wang H.T. (2010). Summarization of magnetorheological fluid and its application. J. Acad. Armored Force Eng..

[4] Fu J., Bai J.F., Lai J.J., Li P.D., Yu M., Lam H.K. (2019). Adaptive fuzzy control of a magnetorheological elastomer vibration isolation system with time-varying sinusoidal excitations. J. Sound Vib..

[5] Brigadnov I.A., Dorfmann A. (2005). Mathematical modeling of magnetorheological fluids. Continuum Mech. Thermodyn..

[6] Jolly M.R., Carlson J.D., Muñoz B.C. (1996). A model of the behavior of magnetorheological materials. Smart Mater. Struct..

[7] Varela-Jiménez M.I., Luna J.L.V., Cortés-Ramírez J.A., Song G. (2015). Constitutive model for shear yield stress of magnetorheological fluids based on the concept of state transition. Smart Mater. Struct..

[8] Pan S., Wu J.Y., Hu L., Shen F., Sun M., Zhou L.W. (1997). Yield stress and temperature effect of magneto-rheological fluids. Funct. Mater..

[9] Cremer P., Hartmut L., Andreas M.M. (2016). Superelastic stress–strain behavior in ferrogels with different types of magneto-elastic coupling. Phys. Chem. Chem. Phys..

[10] Anupama A.V., Khopkar V.B., Kumaran V., Sahoo B. (2018). Magnetic field dependent steady-state shear response of Fe_3_O_4_ micro-octahedron based magnetorheological fluids. Phys. Chem. Chem. Phys..

[11] Tao R., Jiang Q. (1998). Structural transitions of an electrorheological and magnetorheological fluid. Phys. Rev. E.

[12] Gao C.F., Zhang G., Chen W.Z., Ji H.F., Ren S.Q., He X.S. (2015). Temperature dependence of shear yield stress of magnetorheological fluid in inhomogeneous complex field. J. Magn. Mater. Devices.

[13] Chen W., Shi L.Y., Zhang G., He X.S., Gan J.F. (2016). Establishment and error analysis on a theoretical model for the transfer of the fluid layers in the shear mode. J. Magn. Mater. Devices.

[14] Ma L., Song W.L., Wang R.S., Xiu S.C. (2017). Study on shear stress model of magnetorheological fluids with distance weighted factors. Smart Mater. Struct..

[15] Lee S., Shin K.Y., Jang J. (2015). Enhanced magnetorheological performance of highly uniform magnetic carbon nanoparticles. Nanoscale.

[16] Ruan Z.W. (2006). The Theoretical Research on the Shear Stresses of Magnetorheological Fluids Based on BCT Model. Master’s Thesis.

[17] Li H.T., Peng X., Chen W. (2005). Simulation of the chain-formation process in magnetic fields. J. Intell. Mater. Syst. Struct..

[18] Zhang X.D. (2016). Monte Carlo Simulation of Magnetorheological Liquid Phase Separation. Master’s Thesis.

[19] Li H.T., Peng X.H., He G.T. (2010). Research status of mechanism and behavior description of magnetorheological fluids. Mater. Rev..

[20] Melle S., Calderón O.G., Rubio M.A., Fuller G.G. (2002). Rotational dynamics in dipolar colloidal suspensions, video microscopy experiments and simulations results. J. Non-Newton. Fluid Mech..

[21] Mohebi M., Jamasbi N., Liu J. (1996). Simulation of the formation of nonequilibrium structures in magnetorheological fluids subject to an external magnetic field. Phys. Rev. E Stat. Phys. Plasmas Fluids Relat. Interdiscip. Top..

[22] Wu W.Y. (1983). Fluid Mechanics.

[23] Zhang Y. (2007). Fundamentals of Computational Material Science.

[24] Zhu Y., Gross M., Liu J. (1996). Nucleation theory of structure evolution in magnetorheological fluid. J. Intell. Mater. Syst. Struct..

[25] Lópezlópez M.T., Kuzhir P., Lacis S. (2006). Magnetorheology for suspensions of solid particles dispersed in ferrofluids. J. Phys. Condens. Matter.

[26] Bossis G., Lacis S., Meunier A., Volkova O. (2002). Magnetorheological fluids. J. Magn. Magn. Mater..

[27] Ren S.Q. (2015). Research on MRF Shear Characteristics of the Non-Uniform Magnetic Field and Temperature Composite Field. Master’s Thesis.

[28] Chen B.S., Huang Y.J. (2009). Application of fractional calculus on the study of magnetorheological fluids’ rheological characterization. J. Huaqiao Univ. Nat. Sci..

[29] Zhao K.H., Chen X.M. (1985). Electromagnetism.

[30] Li D.C. (2003). Theory and Application of Magnetic Fluids.

[31] Guo C.W., Chen F., Meng Q.R., Dong Z.X. (2014). Yield shear stress model of magnetorheological fluids based on exponential distribution. J. Magn. Magn. Mater..

[32] Feng M.F. (2016). Research on the Constitutive Property of Magneto-Rheological Fluid and its Application on a Hydraulic Bush. Master’s Thesis.

[33] Ivaneyko D., Toshchevikov V., Saphiannikova M., Heinrich G. (2012). Effects of particle distribution on mechanical properties of magneto-sensitive elastomers in a homogeneous magnetic field. Condens. Matter Phys..

[34] Zhu Y.S., Gong X.L., Li H., Zhang P.Q. (2006). Numerical analysis of shear yield stress of magnetorheological fluid. J. China Univ. Min. Technol..

[35] Ashtiani M., Hashemabadi S.H., Ghaffari A. (2015). A review on the magnetorheological fluid preparation and stabilization. J. Magn. Magn. Mater..

